# Leveraging artificial intelligence for the management of preschool wheeze: A narrative review

**DOI:** 10.1111/pai.70207

**Published:** 2025-10-01

**Authors:** Anglin Dent, Mohammad Kaviul Khan, Padmaja Subbarao

**Affiliations:** ^1^ Institute of Health Policy, Management and Evaluation, Dalla Lana School of Public Health University of Toronto Toronto Ontario Canada; ^2^ Temerty Faculty of Medicine University of Toronto Toronto Ontario Canada; ^3^ Program in Translational Medicine SickKids Research Institute Toronto Ontario Canada; ^4^ Division of Respiratory Medicine, Department of Pediatrics The Hospital for Sick Children Toronto Ontario Canada; ^5^ Department of Physiology & Dalla Lana School of Public Health University of Toronto Toronto Ontario Canada

**Keywords:** artificial intelligence, asthma, personalized medicine, preschool, wheeze

## Abstract

Management of preschool wheeze is notoriously challenging given heterogeneous clinical trajectories and underlying biological mechanisms dictating therapeutic response. Data‐driven approaches have highlighted the value of identifying individual wheeze phenotypes and underlying biomarkers to support a personalized management approach; however, these advancements have yet to be translated into clinical management. Here, we discuss key opportunities for Artificial Intelligence and Machine Learning to support personalized approaches to wheeze management through vast pattern‐recognition capabilities. Advancements in the development of tools for objective symptom evaluation, remote symptom monitoring, and prediction of clinical trajectories are summarized. Key considerations for the responsible and successful deployment of such promising technologies in real‐world clinical settings are emphasized, including prevention of algorithmic biases, promotion of prediction transparency, and establishing standards for patient data privacy and equitable access to novel technologies.

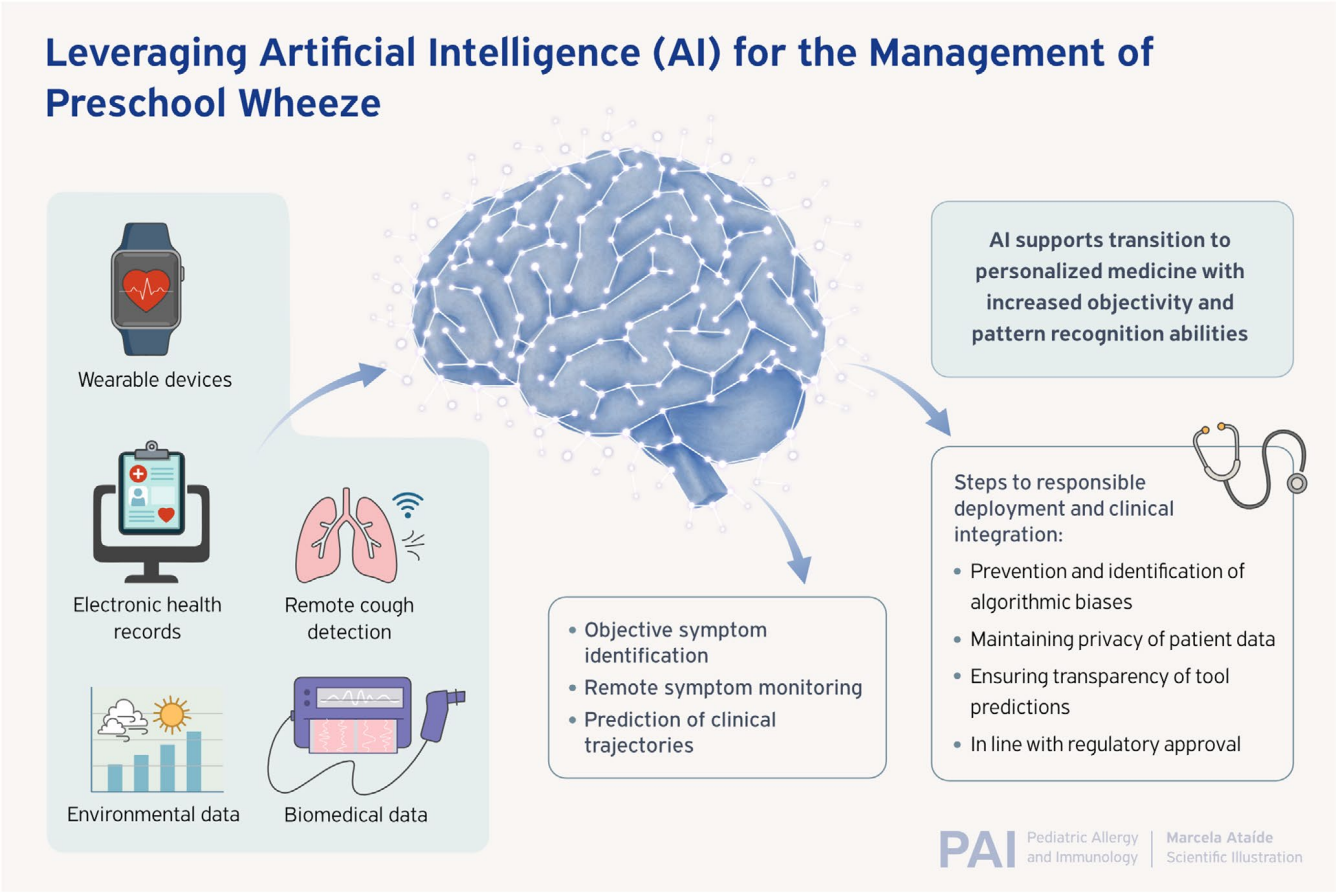

**Key message**

With comprehensive evaluation and carefully considered deployment approaches, artificial intelligence and machine learning‐based technologies have the potential to improve the management of preschool wheeze through objective evaluation of patient symptoms and identification of biomarkers and risk factors for prediction of pertinent patient outcomes.

## 1 THE CHALLENGE OF EARLY CHILDHOOD AND PRESCHOOL WHEEZING

Wheezing, a musical, high‐pitched, and continuous sound emitted from the chest during exhalation^1^ is a common objective childhood symptom^2^ representing respiratory morbidity. In fact, approximately 50% of children will report at least one episode of wheeze in their first year of life.^3^ Recurrent wheeze (1+ documented objective confirmation of wheeze) occurs in approximately one third of preschool‐aged children.^4^ Multiple longitudinal cohorts using agnostic data‐driven techniques all describe a “persistent wheeze” phenotype pattern of early life onset wheezing that is highly associated with an asthma diagnosis.^5^, ^6^, ^7^, ^8^, ^9^ However, these data‐driven approaches have not been translatable to clinic in a prospective fashion. Recently, an expert panel has suggested a standardized definition for “preschool wheeze”^10^ that acknowledges the growing body of literature linking early life wheezing (whether transient or not) with long‐term pulmonary sequelae and aims to facilitate future research studies of preschool wheezing syndromes based on “treatable traits”.^11^ Management of preschool wheeze in the early childhood or preschool population is a significant healthcare concern due to the substantial burden at both the patient level and healthcare systems level, with asthma representing the most common chronic disease in children.^12^

Preschool wheeze significantly impacts an affected child's daily life. As described by Oostenbrik et al. (2006), the impact of wheeze on children's physical functioning decreases reported quality of life, as well as perceived well‐being, functional status, emotion, and general health.^13^, ^14^, ^15^ Persistent wheeze commonly disrupts sleep, hindering children's ability to fall asleep and causing frequent disruptions overnight.^16^ Such disruptions are implicated in observed emotional and behavioral co‐morbidities among children with persistent wheeze, including a high prevalence of anxiety, depression, aggressive behaviors, and attentional challenges that persist into adulthood.^17^ Preschool wheeze additionally impacts a child's ability to attend school, with reports indicating 59% of affected children experience asthma‐related school absenteeism.^18^ Further, early life preschool wheeze is associated with long‐term lung health conditions and decreased lung function that persists into adulthood.^19^ Ultimately, the impact of preschool wheeze on the healthcare system is significant and a serious burden, as the leading cause of hospitalizations and emergency department (ED) visits for preschool‐aged children.^20^

The high prevalence of wheeze in infancy, often resolved with child growth, is due to the collapse and subsequent obstruction of the intrathoracic airway occurring with high lung compliancy in infancy and the inward pressure of expiration.^21^ However, it is unclear how and why wheezing remains a problem in a subset of the population beyond infancy. Though key predictive demographic, genetic, and environmental factors for wheeze persistence, including male sex, ethnicity, lower socioeconomic status, air pollution, climate differences, respiratory viral infections, allergens, maternal smoking, and maternal nutrition^10^, ^22^, ^23^, ^24^ have been identified, individual patient presentations and illness trajectories are challenging to predict and there is no single indicator of which patients will go on to develop preschool wheeze. Further, presentations of preschool wheeze may be split into multiple phenotypes, representative of age of onset, symptomatic patterns, lung function measures, and airway responsiveness.^22^, ^23^, ^25^ Phenotypes have previously been related to specific physiologic characteristics, such as IgE‐associated wheeze, in which patients with an allergic asthma phenotype have a demonstrated increase in levels of the allergic inflammation marker IgE correlated with airway hyper‐responsiveness to methacholine.^26^, ^27^ Underlying these distinct clinical presentations are also distinct mechanistic pathways, known as asthma endotypes.^28^ Consequently, there has been advocacy for treatment based on “treatable traits” of lung function and inflammation. This approach is challenged in children of preschool age, where trait measurement is complicated by a lack of standardized methodology for their clinical assessment.

In addition to identifying individual wheeze phenotypes and underlying mechanisms, effective management of wheeze also relies on accurate detection and reporting. When patients and caregivers seek medical attention relating to preschool wheeze, patient management often relies on parental reporting of symptoms, with varying accuracy.^29^ Even among clinicians, inter‐rater agreement for wheeze identification varies significantly.^30^ Further, clinicians and caregivers may be biased toward labeling respiratory symptoms as “wheeze”. Thus, accurately identifying wheeze within the preschool population and correctly attributing respiratory symptoms to the appropriate medical condition is critical to ensure both adequate management of patient symptoms and correct allocation of therapeutic resources.

## 2 ADVANCES IN THE UNDERSTANDING OF PRESCHOOL WHEEZE

Though the management of preschool wheeze remains challenging, significant strides have been made to improve our understanding of the biology of the heterogeneous wheeze syndromes. This has largely resulted from data‐driven analysis of physiological data spanning blood eosinophilic levels, inflammatory markers, lung function, microbiome data, genetic risk scores, and patient exposures.^31^, ^32^, ^33^, ^34^, ^35^ For example, Fitzpatrick et al. (2016) highlighted the utility of positive aeroallergen sensitization and peripheral blood eosinophil counts as markers for predicting preferential patient response to daily inhaled corticosteroids.^36^ The development of tools to predict treatment response and preschool wheeze heterogeneity has the potential to improve outcomes and quality of life for responders while minimizing adverse medication effects among non‐responders.^37^, ^38^ Furthermore, assessments that provide insight into the biology associated with non‐response offer a discovery pathway for novel therapeutics for high‐burden preschool wheezing children. Further insights into unique markers of distinct wheeze trajectories that highlight differences in biology may provide novel pathways for early personalized protocols for asthma management, ultimately preventing associated morbidity and impacting long‐term wheeze trajectories.^39^

## 3 CURRENT APPROACHES TO SCREENING FOR PRESCHOOL WHEEZE

Nearly 50% of children will experience wheezing in the first year of life, and most will resolve fully after their first episode. Identifying the subset of preschool wheezing children who are “at risk” for repeated attacks, morbidity, and long‐term pulmonary function sequelae is of critical importance to identify for interventions. As such, there are several screening tools that vary in their predictive capacity (sensitivity, specificity, positive predictive value, and negative predictive value) and predictor variables. Ideal features for the predictor variables would be those that are easily acquired in a low‐resource setting and applicable in the general population, given the prevalence of preschool wheezing.

Screening tools such as the Persistent Asthma Predictive Score (PAPS),^40^ the Asthma Predictive Index and its modified version (API and mAPI),^41^, ^42^ and the Pediatric Asthma Risk Score (PARS)^43^ leverage identified risk factors from association studies and incorporate measurements such as eosinophilia levels and aeroallergen sensitization. Though these tools demonstrate strong predictive power, the inclusion of measures obtained through invasive tests limits their utility in the general population. Other screening tools address such limitations by incorporating only clinically available metrics, such as the Childhood Asthma Risk Tool (CHART),^44^ the Prevention and Incidence of Asthma and Mite Allergy (PIAMA) risk score,^45^ and the Predicting Asthma Risk in Children (PARC) tool.^46^ An additional consideration for the utility of such tools is the populations from which they were developed and their ability to generalize to separate populations. Many screening tools utilized high‐risk cohorts to develop their tools with limited validation in the general population, with the exception of PARS and CHART. This limitation was illustrated in a recent prospective validation of the PARC tool, in which Berger et al. (2022) identified that though powerful among their original cohort, performance was significantly limited when externally validated in a clinical cohort.^47^ Furthermore, the incorporation of patient race in asthma screening algorithms (e.g., PARS) is highly controversial. Given the absence of genetic data validating race‐based genetic risks of asthma, prediction of asthma based on patient race is heavily confounded.^48^, ^49^ It must be considered that patient race may be representative of other environmental, social, and psychological risk factors for asthma known to vary among race, ethnicity, and social classes.^50^ Thus, in the consideration of developing screening tools for preschool wheeze, it is critical that such tools are developed from representative populations, undergo extensive external validation, and employ responsible integration of known risk factors to ensure such tools are not at risk of deepening systemic differences in quality and access to care.

## 4 ARTIFICIAL INTELLIGENCE TO FACILITATE MANAGEMENT OF PRESCHOOL WHEEZE

Perhaps the most powerful recently available tool to adequately manage preschool wheeze heterogeneity is Artificial Intelligence (AI). Broadly, AI refers to the concept of computationally replicating human intelligence, including machine learning (ML). ML involves the use of algorithms to process vast quantities of complex data, identify and learn patterns within such datasets, and subsequently draw conclusions from learned observations and patterns.^51^ Machine learning can further be broken down into supervised and unsupervised learning tasks. In supervised learning, predictions are made based on data with labeled outcomes. For example, in a dataset which consists of images of labeled dogs and cats, the machine learning tool will learn each label and will label a previously unencountered image as either a dog or cat based on its learnings from the original training set. Alternatively, unsupervised learning involves the grouping of unlabeled input data based on the identification of shared patterns within the groupings. For example, if the dataset of cats and dogs were not labeled and processed by the machine learning tool, the tool would discover patterns within the data itself which distinguish dogs from cats (e.g. cat whiskers, pointed ears, etc.) and create clusters based on the similarities and differences of the characteristics of each image.^52^, ^53^ In the context of preschool wheezing, AI and ML approaches are capable of efficiently and powerfully identifying themes within vast quantities of medical data to delineate heterogeneous presentations and mechanisms of the condition. Coupled with the rapid expansion of biomedical data collection, including electronic medical record notes, medical imaging, laboratory values, and physiological data collected through wearable devices, ML technologies have provided a novel approach to identify pertinent patterns among wheezing biomarkers and risk factors for childhood wheezing conditions.

Additionally promising for the outlook of comprehensive wheeze management is the ability for ML technologies to serve as clinical decision support tools. As described, there is a significant component of subjectivity in the identification, classification, and management of childhood wheeze. The use of ML to standardize symptom monitoring and management approaches has the potential to improve and standardize care for all childhood wheezers, irrespective of proximity to respirology experts and age limitations for objective spirometry measurements.^54^ Further, tools which can monitor and classify symptoms remotely may allow for increased caregiver awareness of symptom control and alerts for signs of wheeze exacerbation, in addition to supporting efforts to promote early identification and screening of populations at risk for persistent wheeze. Such ML‐based approaches have the potential to support proactive management of symptoms among high‐risk patients and ultimately reduce the burden of wheeze on healthcare systems (e.g. ED visits and hospitalizations).^55^

## 5 METHODS

To further illustrate the potential for AI and ML approaches to address major challenges in the management of early childhood wheeze, we conducted a scoping review of literature spanning from 1987 to 2025 on PubMed and MEDLINE Ovid databases. Keywords used in the search criteria included: “preschool wheeze”, “asthma”, “pediatrics”, “machine learning”, “artificial intelligence”, “artificial intelligence ethics”, and “responsible artificial intelligence”. Reference lists of retrieved studies were also consulted. In total, 508 studies were identified in the initial searches, and 158 were reviewed following abstract and full‐text screening. Referencing the findings of these studies, we summarize the advancements in the application of AI and ML in the management of persistent wheeze, in addition to providing recommendations to ensure the fair and safe application of such tools.

## 6 AI FOR REMOTE SYMPTOM MONITORING

AI for remote symptom monitoring supports a system for the standardized assessment of wheeze control by caregivers. To date, multiple studies have developed ML algorithms capable of identifying when wheeze symptom control is at risk of deteriorating, as compared to physician clinical assessment or completion of validated patient‐reported symptom control questionnaires.^56^, ^57^, ^58^ One simple approach to this challenge has been the application of an AI‐based analysis of breath sounds, through recordings or at‐home stethoscopes, so that caregivers may accurately characterize an asthma exacerbation without the need for physician assessment.^59^ Emeryk et al. demonstrated the utility of such an approach for characterizing symptom exacerbations in children younger than 5 years of age, who are the largest contributors to wheeze‐related emergency department visits and hospitalizations.^20^, ^59^ By combining multiple physiologic (e.g., heart rate and respiratory rate) and pathologic lung sound variables collected by an AI‐assisted stethoscope, their tool was capable of identifying asthma exacerbations more accurately than subjective assessment by parents or single‐parameter vital sign measurements, when compared to expert clinician reference standards. The predictive power of this tool was improved further with the combination of parental reports and objective physiological measurements such as peak expiratory flow and peripheral capillary oxygen saturation. With the inclusion of novel technologies such as smart wearable devices^57^ and digital twin systems,^60^ there is potential for a continuous stream of patient physiological data or environmental measures (e.g., pollutants, pollens, and weather conditions) to be integrated and assessed by ML technologies for the prediction of exacerbation risk. Indeed, Hosseni et al. demonstrated the utility of a random forest classifier to project a patient's risk level with an accuracy of 80.10% using physiological data collected from a wearable device and environmental data collected from dust and particulate matter sensors.^57^

## 7 AI FOR STANDARDIZED SYMPTOM EVALUATION

As described, assessment of wheeze by clinicians is at risk of subjective assessment. To date, a wide range of AI and ML technologies which aim to improve the clinical assessment and characterization of wheeze have been developed. These include algorithms which attempt to standardize the identification of wheeze and wheeze sub‐types (e.g. bronchial) through traditional auscultation, including in patients as young as 1 month.^61^, ^62^, ^63^, ^64^ Deep Breath, developed by Heitmann et al. (2023), leveraged a combined convolutional neural network and logistic regression classifier approach to identify wheeze with an Area Under the Receiving Operator Curve (AUROC) of 0.91 and externally validated the tool using datasets from 3 different countries.^65^ Beyond auscultation, AI technologies have been applied to standardize more technical analyses for wheeze and asthma status, including Forced Oscillation Technique (FOT) measurement and chest Computed Tomography (CT) evaluation.^66^, ^67^

## 8 AI PREDICTIONS OF AT‐RISK POPULATIONS FOR PERSISTENT WHEEZE AND RELATED OUTCOMES

As described, the field of pediatric asthma has made vast advancements in the identification of environmental, genetic, and exposure risk factors predictive of disease and symptom burden. The pattern prediction capabilities of ML technologies have advanced this field of work even further, with many research groups leveraging ML algorithms to identify underlying relationships among biomarkers and exposures of individual patients to enhance the identification of populations at greatest risk of persistent wheeze. These include the use of prenatal, perinatal, postnatal, and environmental factors, such as early‐life air toxicities^68^, ^69^; as well as known sociodemographic risk factors for asthma including patient race and ethnicity.^70^ The power of combining ML with large datasets was demonstrated by He et al. (2024), who leveraged ML tools to analyze data from the longitudinal CHILD birth cohort study. Here, the research team utilized a dataset consisting of 132 variables, spanning parental medical history, maternal psychological health, clinical characteristics of the child (e.g. anthropometrics, sex, gestational age, presence of atopy, wheeze status), and environmental exposures (e.g. home environment, antibiotic exposure, breastfeeding history) from 6 different time points. Multiple ML models were built from this dataset in the search for the best model to predict asthma diagnosis. Ultimately, the authors found that a combination of ML approaches, such as random forest and logistic regression, with the longitudinal CHILD data supported the identification of patients at risk of persistent wheeze with an AUROC greater than 0.90. Factors such as maternal asthma, antibiotic exposure, and history of lower respiratory tract infections were identified as the most predictive variables for asthma diagnosis.^71^

ML‐based prediction models span beyond simply predicting an asthma diagnosis, with multiple research teams striving to generate models predictive of distinct asthma phenotypes. For example, approaches that have leveraged ML analysis of breath samples collected from *eNoses* have been able to distinguish between non‐atopic and atopic wheeze among a cohort of individuals with wheeze.^72^ Other ML‐based clustering approaches have revealed previously unidentified clinical phenotypes through ML analysis of asthma outcome data (e.g., treatment response and hospitalization outcomes), as well as laboratory biomarkers.^73^, ^74^, ^75^ For example, Wu et al. (2014) assessed 112 clinical (e.g., family history of asthma, medication use, healthcare utilization), physiologic (e.g., lung function measurements), and inflammatory (e.g., eosinophilia, white blood cell and neutrophil count) variables collected from a cohort of patients with persistent wheeze using unsupervised learning approaches. Through identification of underlying patterns among these variables between patients, this technique revealed 6 clusters of patients with distinct physiologic and inflammatory profiles, as well as distinct illness trajectories (such as early vs. late‐onset asthma, medication use, quality of life, and repeat healthcare utilization). Such findings highlight the potential of ML to assist with data‐driven approaches to further personalized medicine strategies for managing patients with persistent wheeze, such as by helping to triage patients in acute settings and select the most appropriate therapies for individual patients.

ML approaches have additionally been used to build prediction models for outcomes of persistent wheeze, including exacerbations,^76^, ^77^ emergency department visits,^78^, ^79^ and hospitalizations due to asthma.^80^, ^81^ Such models may be beneficial for both patients and caregivers to understand the progression of their own symptoms and when to implement treatment, in addition to providing benefits to the healthcare system by forecasting the clinical burden of asthma in a specific hospital or emergency department. For example, Moustris et al. (2011) developed an ML‐based approach to predict the number of hospital admissions for asthma 7 days in advance, through leveraging robust, real‐time environmental datasets of meteorological data and ambient air pollution.^82^ Though the majority of such tools demonstrate promise in proof‐of‐concept studies, clinical validation studies are needed to demonstrate their true clinical potential. Studies such as Seol et al. (2021), in which an asthma therapeutic workflow leveraging an ML‐based tool for exacerbation prediction was assessed through a randomized clinical trial, highlight the potential for promising models to offer minimal benefits when deployed in real‐world settings. For example, this tool identified no difference in control of asthma, despite leveraging a promising ML‐based prediction model for clinical outcomes.^83^

## 9 CONSIDERATIONS FOR THE RESPONSIBLE USE OF AI IN MANAGEMENT OF WHEEZE

With such promising developments in the field of AI and ML for the management of wheeze, significant considerations regarding the responsible use of applying these tools in real‐world clinical scenarios must be made – particularly in pediatric populations, where they may help to address the burden of preschool wheeze. These include comprehensive assessment for and prevention of algorithmic biases, evaluating the performance between patient populations and clinical contexts, establishing standards for transparent tool predictions, ensuring standards are established for to maintain the privacy of patient data, and promotion of universal access to the newly developed technologies.

The risk of algorithmic biases has long been identified as a barrier for acceptance of novel AI‐based technologies in healthcare, with significant real‐world examples highlighting the implications of deploying a biased algorithm on a large scale.^84^ Prevention and identification of algorithmic biases should be ongoing in the development of an AI‐based tool. Before development of the tool even begins, ensuring that datasets used for training and testing are representative of the population for which the tool is targeted is critical.^54^ Without representation in the datasets used to train such technologies, these tools have the potential to create or exacerbate existing divides in the standard of care for certain populations. This may be a particular concern for populations in which there is limited health data due to longstanding systemic racism and discrimination in the healthcare system, such as indigenous populations.^85^ Training and testing datasets should be critically evaluated prior to the development of novel technologies to identify limitations proactively. Once identified, developers should consider purposive sampling to ensure that datasets used capture all patients for which the tool is intended to help treat, with the goal of reducing, rather than magnifying, existing barriers in healthcare for specific populations.^86^ Another consideration for reducing the risk of biasing algorithms is limiting the use of proxy variables, or indirect measures, for predicted outcomes of interest. In the context of asthma prediction, medication use (e.g. bronchodilators, inhaled corticosteroids, or oral corticosteroids) is often used as an alternative outcome to asthma, as it may be easier to quantitatively capture. Algorithms which employ such proxy variables may be inherently biased towards prediction of asthma in specific populations with greater access to medical resources or be influenced by differences in prescribing practices between physicians.^87^ Further, in the design of algorithms for use in the pediatric population, particular consideration must be made to prevent “age‐related algorithmic bias”. Pediatric populations should always be represented in the dataset from which the algorithm is trained and generalizations of findings from adult populations should be limited.^88^ This can be challenging, given a frequent under‐representation of individuals under the age of 18 in many research studies.^89^

External and prospective validation studies play a key role in the identification of algorithmic biases which may not be obvious until deployed in a real‐world setting. Such studies allow for the evaluation of how developed algorithms may translate to previously unencountered populations that may not have been captured in original training and testing datasets. Though this has long been considered a critical next step in the development of novel AI‐based solutions for healthcare, this is often the stage in which algorithms fail and their path to clinical integration is stalled.^90^ Additionally, this critical evaluation is often overlooked. In the context of ML for the management of respiratory conditions, a previous study examining the ML tools for predicting chronic respiratory conditions identified that only 1 of the 25 papers included in the review conducted an external validation in a new geographic region.^87^ Ultimately, algorithms built in preparation to encounter differences in real‐world data compared to training data (i.e. dataset shift) will be more likely to perform in external validation and be generalizable to various clinical populations and contexts.^91^ Further clinical validation of a newly developed ML technology may come in the form of a silent trial, in which the tool is deployed in the real‐world clinical setting, but predictions are not disclosed to treating physicians.^92^ To that end, the silent trial does not assess the impact of the tool on patient outcomes but rather evaluates performance of a new tool in comparison to the real‐time clinical course of the patient. This step can often serve as a safety measure, allowing the evaluation of the tool in the setting within which it will be deployed, but not yet influencing patient care. Once external validation studies have been completed and a tool is deployed, continuous evaluation of model performance across patient populations is necessary. As patient populations inherently change over time (i.e. data drift), it is essential to ensure models continue to perform adequately and equally across all encountered patients. Models which employ continuous learning approaches, in which real‐time predictions are scored and fed back to continuously refine the model, may be less likely to experience detrimental changes to performance metrics overtime.^93^

Ensuring transparency of newly developed ML tools is another key component of supporting the responsible use of ML in clinical settings. ML‐based technologies have often failed to be integrated into established clinical workflows and achieve acceptance among clinical end‐users due to their “black box” nature.^94^ The development of algorithms which use a minimal number of clinically relevant predictors will inherently be more interpretable by clinicians and more likely to be accepted for use in a clinical setting. If this is not possible, models may improve transparency and explainability of predictions through the integration of feature attribution methodologies. For example, the use of Shapley additive explanation (SHAP) values allows for users to interpret which predictor variables had the most influence on a model's prediction, providing a high‐level explanation to clinical users on how predictor variables are used to generate a predicted output.^95^ Establishing clinician understanding of how to critically evaluate and what actions to take with a predicted outcome is another crucial consideration in the development and deployment of new ML technologies.^96^ In the context of algorithms for wheezing, predictions must align with available clinical workflows and standards of care. To promote clinician acceptance and integration within current clinical approaches, benefits of integrating the tool should be emphasized. For example, algorithms which predict children at high risk of persistent wheezing should be paired with an established clinical pathway which could enable improved management of this population.

Equally critical is ensuring that novel ML technologies may be deployed in the clinical setting while maintaining patient privacy. Evaluation of how much patient data is necessary to generate a prediction should guide decisions regarding which variables are included in the model, with careful comparison of the risks to patient privacy and the benefits of including sensitive patient information in the model.^97^ Risk evaluations should be conducted prior to deployment and on a regular basis following deployment to ensure patient confidentiality is maintained.^98^, ^99^ This is critical both to ensuring novel technologies are compliant with regulatory standards and improving patient trust in the use of such technologies to manage their conditions.^100^ In the context of preschool wheeze, there are further ethical considerations for deploying new technologies that use pediatric medical data. Though debated, current perspectives suggest that caregivers should be informed about the use of ML‐based technologies in the care of their child and be provided with the option to opt out of such approaches.^101^

Finally, the responsible application of ML approaches to manage preschool wheeze must involve strategies to ensure such technologies decrease, rather than amplify, disparities in access to care. AI and ML‐based technologies have frequently been positioned as opportunities to address longstanding racial and socioeconomic disparities in access to care and specialist resources.^102^ However, ML algorithms have the potential to be biased towards certain populations and may inadvertently base predictions in line with established health disparities.^103^ Further, the deployment of novel technologies in settings with inadequate infrastructure (e.g., training for clinicians, digital resources, financial support) or greater patient barriers (e.g., language barriers, lower health literacy) will be less likely to successfully translate into clinical settings and therefore further exacerbate differences in the care of patients based on community income level and proximity to affluent and academic institutions.^104^ This is particularly relevant in the context of wheeze, as residing in lower income neighborhoods, family income, parental education, and access to care are known predictors of symptom burden.^105^ Thus, in the consideration of how we may integrate such promising technologies into current clinical workflows, targeted approaches involving technological developers, healthcare providers, and policymakers must be utilized to make the technologies accessible to geographically remote and low‐income regions where asthma prevalence is high.

## AUTHOR CONTRIBUTIONS

**Anglin Dent:** Conceptualization; investigation; writing – original draft; methodology; writing – review and editing; formal analysis. **Mohammad Kaviul Khan:** Writing – review and editing. **Padmaja Subbarao:** Conceptualization; investigation; methodology; writing – original draft; writing – review and editing; project administration; supervision; resources.

## FUNDING INFORMATION

P.S. holds a Canada Research Chair Tier 1 in Pediatric Asthma and Lung Health.

## CONFLICT OF INTEREST STATEMENT

The authors declare no commercial or financial conflicts of interest.

## PEER REVIEW

The peer review history for this article is available at https://www.webofscience.com/api/gateway/wos/peer‐review/10.1111/pai.70207.
